# Quantitative comparison of a novel swept-source optical coherence tomography angiography device with three established systems

**DOI:** 10.1038/s41598-025-04650-9

**Published:** 2025-06-20

**Authors:** Michael Hafner, Siegfried G. Priglinger, Bettina von Livonius, Maximilian J. Gerhardt

**Affiliations:** Department of Ophthalmology, LMU University Hospital, Ludwig-Maximilians-Universität München, Mathildenstraße 8, 80336 Munich, Germany

**Keywords:** Optical coherence tomography angiography (OCTA), Swept-source OCTA, Retinal microvasculature, Superficial capillary plexus (SCP), Deep capillary plexus (DCP), Retinal imaging., Retinal diseases, Medical imaging

## Abstract

Optical Coherence Tomography Angiography (OCTA) has become an essential non-invasive imaging technique for high-resolution visualization of retinal microvasculature. This study evaluates the performance of a novel Swept-Source OCTA device, Intalight DREAM, compared to established systems: Heidelberg Spectralis, Topcon Triton, and Zeiss Cirrus. We assessed acquisition time and microvascular parameters in the superficial (SCP) and deep (DCP) capillary plexuses using the OCTA Vascular Analyser algorithm for standardized image analysis across devices on 30 eyes from 15 healthy participants. In the SCP, DREAM demonstrated a higher median vessel length (47 μm) and greater fractal dimension (mean: 1.999) than the other devices, indicating enhanced continuity and network complexity. In the DCP, DREAM showed a smaller foveal avascular zone (median: 0.339 mm^2^) compared to Spectralis (0.51 mm^2^), Triton (0.5935 mm^2^), and Cirrus (0.9145 mm^2^), along with a smaller vessel diameter (median: 23 μm) compared to Triton and Cirrus. With a median imaging time of 9.1 s, DREAM was significantly faster than the Spectralis system (23.3 s) while providing largely comparable image quality, enhancing patient comfort, and potentially minimizing motion artifacts. These findings suggest that DREAM OCT is a promising tool for deep retinal imaging, with strong potential for clinical application and research.

## Introduction

Since its introduction, optical coherence tomography angiography (OCTA) has rapidly become a powerful clinical imaging tool for diagnosing chorioretinal diseases^1^. Unlike traditional methods such as fluorescein angiography and indocyanine green angiography, OCTA is non-invasive and eliminates the need for intravenous dye injections, thereby reducing the risk of allergic reactions, enhancing patient comfort, and making it suitable for a broader patient population^2^. Other potential advantages include rapid acquisition times that facilitate repeated scans, enhanced resolution of capillaries without interference from leakage, and the capability for depth-resolved analysis, allowing for the examination of blood flow at specific axial locations within the retina or choroid^3^.

OCTA functions by detecting changes in reflectivity caused by the movement of red blood cells within blood vessels. It captures these signals from sequential optical coherence tomography (OCT) B-scans, generating motion-contrast images on a pixel-by-pixel basis^4^.

Recently, Intalight Inc. (San Jose, California, USA) introduced a new Swept-Source OCTA (SS-OCTA) system, the DREAM OCT, which is currently approved in China and, just recently, in Brazil^5^. With a scanning rate of 200 kHz, DREAM surpasses the performance of the well-established systems tested in this study (Heidelberg Spectralis: 125 kHz^6^, Topcon DRI-OCT Triton: 100 kHz^7^, and Zeiss Cirrus 5000: 68 kHz^8^).

With the growing variety of OCTA platforms, it is essential to evaluate the consistency of microvascular measurements across devices. Several studies have compared various OCTA machines using different quantitative methods, yielding diverse results regarding the consistency of the analysed parameters^9–12^. For any newly introduced device, it is crucial to understand how its imaging capabilities compare to established systems to ensure that its results are accurate and comparable.

To evaluate the consistency of the DREAM system, we have designed a prospective study aimed at quantitatively comparing this new device with three well-established OCTA platforms: Heidelberg Spectralis OCT module with SHIFT technology (Heidelberg Engineering, Germany), Topcon DRI-OCT Triton Swept-Source OCT (Topcon, Japan), and Zeiss Cirrus 5000 OCT (Zeiss Meditec Inc., Germany). To ensure a standardised cross-device analysis that is as independent as possible from the proprietary image analysis and interpretation software of each device, we employed the OCTA Vascular Analyser (OCTAVA)^13^. This approach allows for a comprehensive comparison of a wide range of image parameters across different systems.

## Methods

Ethics approval was obtained from the Institutional Review Board of the Faculty of Medicine, LMU Munich (study ID: 24–0571), and the study adhered to the principles outlined in the Declaration of Helsinki. Informed consent was provided by all patients. Epidemiological data collected for each patient included age and gender.

The study utilised images captured by four different devices: the Intalight DREAM Swept Source OCT VG200D (Intalight Inc., USA), Heidelberg Spectralis OCT module with SHIFT technology (Heidelberg Engineering, Germany), Topcon DRI-OCT Triton Swept-Source OCT (Topcon, Japan), and Zeiss Cirrus 5000 OCT (Zeiss Meditec Inc., Germany). Scanning rates were 200 kHz for DREAM OCT, 125 kHz for Spectralis OCT, 100 kHz for Triton OCT, and 68 kHz for Cirrus OCT. Scanning laser wavelengths were 1030 –1070 nm (Swept-Source tunable laser) for DREAM OCT, 880 nm for Spectralis OCT, 1050 nm for Triton OCT, and 840 nm for Cirrus OCT^5–8^.

An Automatic Real Time (ART) value of four was applied to every device. ART4 automatically averages four images per scan line, enhancing image quality by reducing noise; decreasing the number of averaged images accelerates the acquisition process. The image resolution for the DREAM OCT and Spectralis OCT was set to 512 × 512 pixels. In contrast, the Triton and Cirrus devices did not offer such high resolution. For these two systems, the highest possible resolution was selected, with 320 × 320 pixels for the Triton OCT and 420 × 420 pixels for the Cirrus OCT, each for the 3 mm × 3 mm scan.

### Participants

Patients were recruited from the Department of Ophthalmology at LMU University Hospital Munich in December 2024 and January 2025. Each participant included in the study had two healthy eyes with no prior diagnosis of chorioretinal disease or history of ocular surgery that could affect the retinal microvasculature. Another exclusion criterion was a history of any systemic disease, such as arterial hypertension, diabetes mellitus, or chronic kidney disease, which can affect the retina.

### Imaging

Consent was obtained from all participants prior to imaging. Both eyes of each subject were scanned using four different OCTA devices during the same visit: the Intalight DREAM Swept Source OCT (Intalight Inc., USA), Heidelberg Spectralis OCT module (Heidelberg Engineering, Germany), Topcon DRI-OCT Triton Swept-Source OCT (Topcon, Japan), and Zeiss Cirrus HD 5000 OCT (Zeiss Meditec Inc., Germany).

Pupillary dilation was not performed before imaging. For the DREAM, Triton, and Cirrus devices, a 3 mm × 3 mm volume scan centered on the fovea was used, while the Spectralis model employed a 10° × 10° scan pattern (approximately 2.9 mm × 2.9 mm)^14^. Imaging time was recorded for all 30 eyes during OCTA acquisition.

En face images of the superficial capillary plexus (SCP) and deep capillary plexus (DCP) were generated for each eye using the default layer segmentation software. The SCP was defined as the region from the inner limiting membrane to the transition between the inner plexiform layer and inner nuclear layer, while the DCP was defined as extending from the boundary between the inner plexiform and inner nuclear layers to the boundary between the outer plexiform and outer nuclear layers^15^. This standardised definition of the SCP and DCP was applied consistently across all four devices to ensure that the resulting en face images were as comparable as possible before further image analysis. Importantly, the default settings of the various OCTA devices employ differing definitions of the SCP and DCP, which can lead to inconsistent and non-representative results if not adjusted. Thus, standardising these parameters was critical to avoid misleading comparisons between devices.

### Image processing

**Fig. 1:**
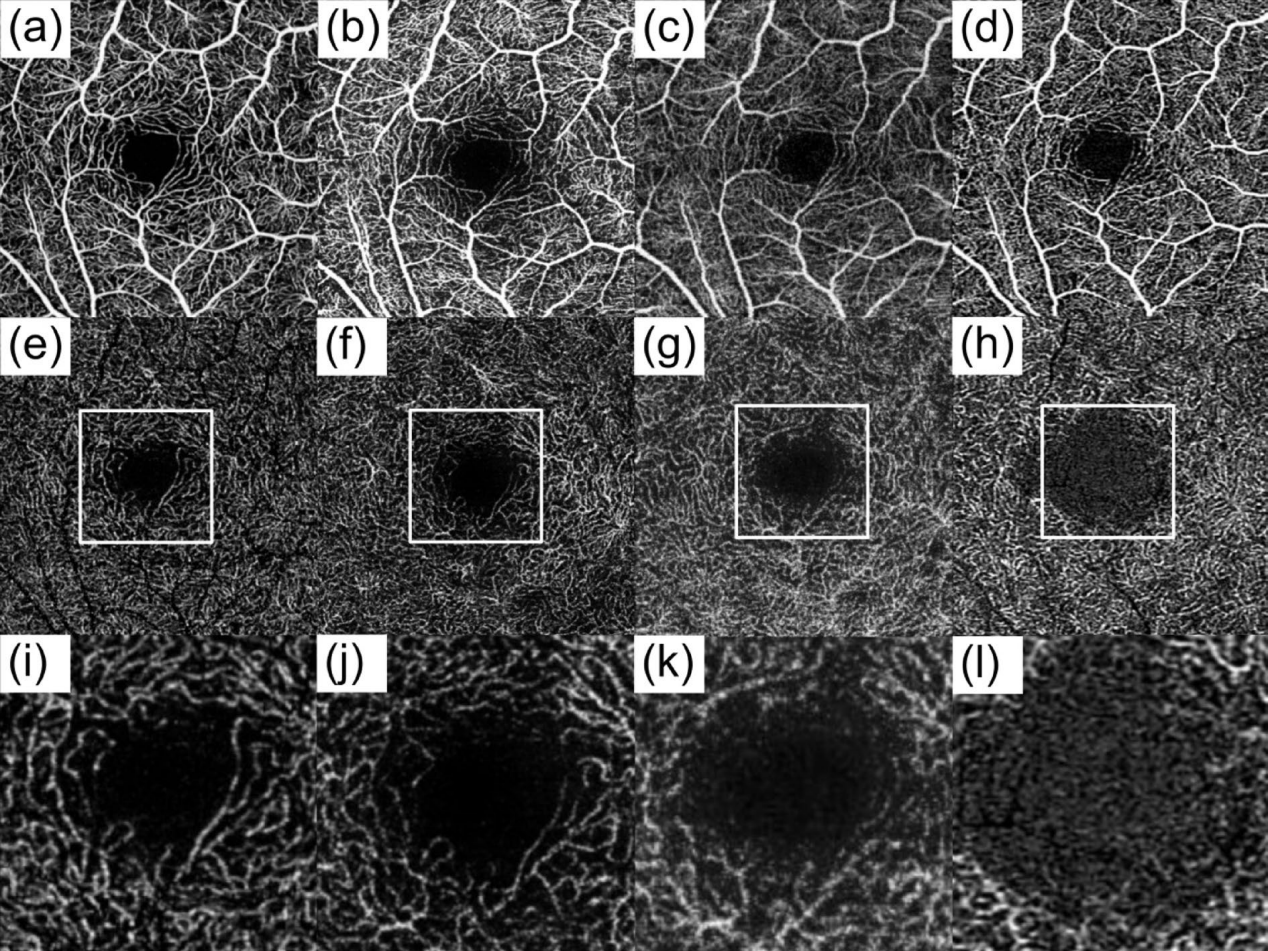
Examples of the OCTA en face images derived from the four different devices (all images obtained from the same patient). First row with images of the superficial capillary plexus from (**a**) DREAM, (**b**) Spectralis, (**c**) Triton, (**d**) Cirrus. Second row with images of the deep capillary plexus from (**e**) DREAM, (**f**) Spectralis, (**g**) Triton, (**h**) Cirrus device. The third row shows an enlarged cutout of the foveal avascular zone and the surrounding area in deep capillary plexus for (**i**) DREAM, (**j**) Spectralis, (**k**) Triton, and (**l**) Cirrus. Notable is the difference in vessel continuity, especially in terms of imaging the foveal avascular zone and surrounding area in the deep vascular plexus.

**Fig. 2:**
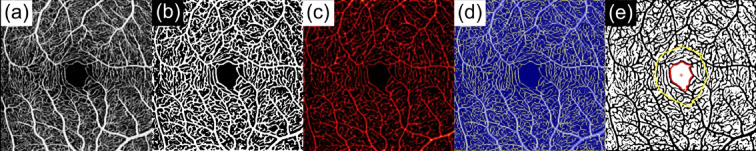
Visualization of the different steps of the OCTAVA image analyzer. (**a**) Resized en face image from DREAM OCTA (512 × 512). (**b**) Image after application of the Frangi filter and binarization. (**c**) Graphical representation of the heatmap of vessel diameter created using a Euclidean distance transform. (**d**) Image skeletonized after application of a MATLAB 3D thinning algorithm. (**e**) Graphical result of automated measurement of foveal avascular zone (marked in red; yellow: starting edges of the algorithm).

The Fiji image processing software (National Institutes of Health, MD, USA) was employed for image adaptation. Since the en face images provided by the different devices varied in pixel size, all images were resized to 512 × 512 pixels, which corresponds to the maximum resolution supported by both the DREAM OCT and Spectralis OCT. Examples of resized en face images before further analysis are shown in Figs. 1 and 2a (DREAM OCTA device).

### Image analysis

After resizing, the images were analysed using the OCTA Vascular Analyser (OCTAVA), a tool recently enhanced by Untracht et al.^13^. OCTAVA is an open-source application designed to support the analysis of retinal OCTA images from multiple devices. The software runs within the MATLAB environment, and for this study, it was executed on MATLAB for macOS R2024b (24.2.0.2712019, 64-bit, maca64; MathWorks, Natick, USA).

A two-dimensional Frangi filter (threshold value of three) was applied to identify blood vessels in the en face images. This filter is widely used in angiography as it minimises intensity variations along vessels and suppresses background noise, enhancing image segmentation. The resizing of the images was essential to ensure consistent application of the Frangi filter across datasets from each OCTA device, enabling uniform image analysis and comparison^13^.

The pre-processed image was then segmented into the two categories (i) vessels and (ii) not vessels using fuzzy thresholding (AT kernel size of 70)^16^. The result after applying the Frangi filter and binarization is shown in Fig. 2b.

Post-segmentation, the binarized image undergoes skeletonization via a Matlab 3D thinning algorithm^17^, and a heatmap of vessel diameter is created using a Euclidean distance transform^18^. Network connectivity is analysed by converting the skeletonized image into an undirected graph structure, where branch points are identified, and vessels are classified based on connectivity. Isolated elements and branches below a certain length (twig size) were considered noise and excluded from analysis^13^. Twig size was manually adjusted after visual inspection to a value of two. An example of a heatmap of vessel diameter is presented in Fig. 2c, an example of a skeletonized en face image in Fig. 2d.

Foveal avascular zone (FAZ) is further automatically segmented from the binarized image, and the FAZ area is calculated^13^. An example of automatically segmented FAZ is shown in Fig. 2e.

The following microvascular metrics were analysed cross-device using OCTAVA: vessel area density (VAD), area of the foveal avascular zone (FAZ), total vessel length (TVL), number of nodes, and fractal dimension (FD) for both the SCP and DCP. Additionally, median vessel length (MVL) was analysed for the SCP, while mean vessel diameter (MVD) was assessed for the DCP.

### Data analysis and statistics

Data management was performed using Microsoft Excel Version 16.78.3 for Mac, and statistical analyses were conducted with GraphPad Prism for macOS Version 10.3.1. A significance level of *p* < 0.05 was applied. Biomarker differences across devices were compared using the Friedman ANOVA test. Post-hoc analysis was performed using Dunn’s multiple comparisons test.

To complement p-value-based significance testing, effect sizes were calculated using Cliff’s Delta for paired samples. In paired data, Cliff’s Delta quantifies how often the measurement from the DREAM OCTA device is greater than, less than, or equal to the corresponding measurement from the comparator device. It is calculated as $\delta = {{n_{ + } - n_{ - } } \mathord{\left/ {\vphantom {{n_{ + } - n_{ - } } n}} \right. \kern-\nulldelimiterspace} n}$ where $\:{n}_{+}$ is the number of pairs where the DREAM measurement is greater than the comparator, $\:{n}_{-}$ is the number of pairs where the DREAM measurement is smaller than the comparator, $\:n$ and is the total number of paired observations.

Values of δ range from − 1 (all DREAM measurements smaller) to + 1 (all DREAM measurements larger). A value of 0 indicates no systematic difference. The calculations were performed using RStudio for Mac (2024.12.1 + 563).

## Results

### Baseline demographics

A total of 30 eyes from 15 healthy participants were imaged using four different OCTA devices. To minimise potential bias from image selection, all acquired images were included in the analysis. The participants had an average age of 26.41 ± 2.94 years (mean ± standard deviation), with nine female and six male participants.

### Imaging time

**Fig. 3:**
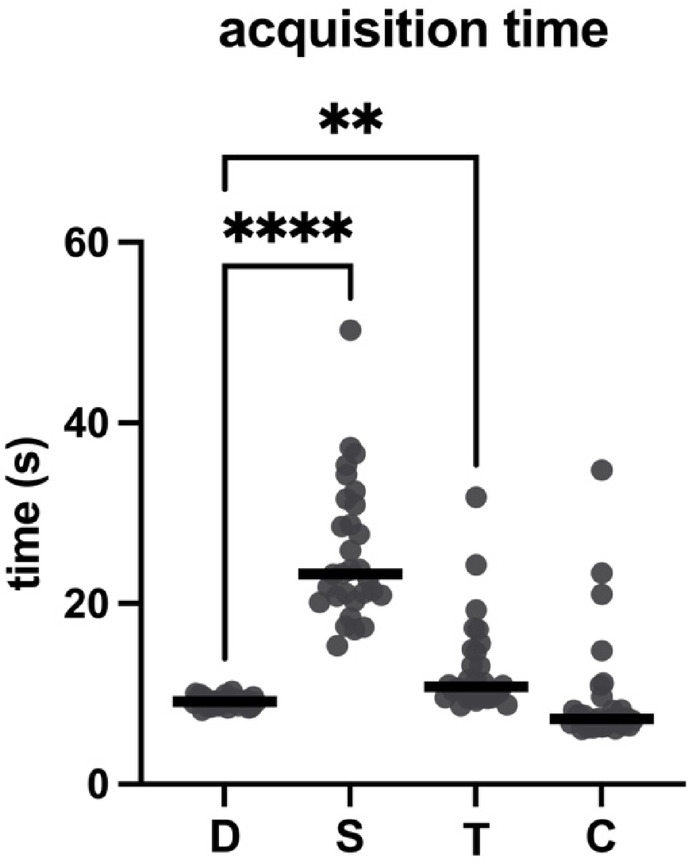
Comparison of image acquisition time for 3 mm × 3 mm OCTA imaging on the different devices. Significant *p*-values (< 0.05) of pairwise comparison with DREAM OCTA are marked by asterisks (with **** as *p* < 0.0001; ** as *p* < 0.01). D: DREAM; S: Spectralis; T: Triton; C: Cirrus.

Imaging time was recorded for all 30 eyes during OCTA acquisition. The median imaging times were as follows: 9.125 s (IQR: 0.805 s) for DREAM OCTA, 23.25 s (IQR: 10.43 s) for Spectralis OCTA, 10.77 s (IQR: 5.132 s) for Triton OCTA, and 7.21 s (IQR: 2.052 s) for Cirrus OCTA. Imaging time for the DREAM OCTA was significantly shorter compared to the Spectralis OCTA (*p* < 0.0001; δ = -1.00) and Triton OCTA (*p* = 0.0082; δ = -0.33). Cirrus OCTA tended to offer smaller median acquisition times without exhibiting a statistically significant difference compared to DREAM. Data is reported in Table 1 and graphically in Fig. 3.

**Table 1:** Acquisition time and interquartile range (IQR) for 3 mm × 3 mm OCTA imaging on the different devices.

	DREAM	Spectralis	Triton	Cirrus
Time (s)	9.125	23.25	10.77	7.21
IQR_time_ (s)	0.805	10.43	5.132	2.052
*p*	–	**< 0.0001**	**0.0082**	> 0.9999
δ	–	− 1.00	− 0.33	0.37

*P*-value is given for pairwise comparison with the novel DREAM OCTA system. Effect size is given with Cliff’s delta (δ).

Significant *p*-values (*p* < 0.05) are reported in bold.

### Superficial capillary plexus

For vessel area density (VAD), the values were measured as follows: 40.2% (IQR: 3.50%) for DREAM OCTA, 44.45% (IQR: 3.30%) for Spectralis OCTA, 30.3% (IQR: 3.59%) for Triton OCTA, and 39.71% (IQR: 5.22%) for Cirrus OCTA. When comparing DREAM OCTA with the other devices, Spectralis OCTA showed a significantly higher VAD (*p* = 0.0019; δ = − 0.87), while Triton OCTA displayed a significantly lower VAD (*p* < 0.0001; δ = 1.00). No statistically significant difference in VAD was observed between DREAM and Cirrus OCTA.

For the foveal avascular zone (FAZ) size, DREAM OCTA showed a median of 0.298 mm^2^ (IQR: 0.164 mm^2^), Spectralis OCTA 0.416 mm^2^ (IQR: 0.1522 mm^2^), Triton OCTA 0.299 mm^2^ (IQR: 0.124 mm^2^), and Cirrus OCTA 0.2845 mm^2^ (IQR: 0.1575 mm^2^). A significant difference was found between DREAM and Spectralis OCTA (*p* < 0.0001; δ = − 0.77), but no significant differences were detected between DREAM OCTA and the other two devices.

For total vessel length (TVL), DREAM OCTA presented a median of 165.2 mm (IQR: 13.7 mm), Heidelberg OCTA 179.2 mm (IQR: 15 mm), Triton OCTA 122.8 mm (IQR: 18.9 mm), and Cirrus OCTA 163.6 mm (IQR: 25.2 mm). DREAM OCTA showed a significantly larger TVL compared to Triton OCTA (*p* < 0.0001; δ = − 0.60), while no significant differences were observed in comparison with Spectralis or Cirrus OCTA.

Regarding the number of nodes in the vascular architecture, DREAM OCTA detected a median of 1403 nodes (IQR: 228), Spectralis OCTA 1711 nodes (IQR: 259), Triton OCTA 729.5 nodes (IQR: 227), and Cirrus OCTA 1382 nodes (IQR: 316). Spectralis OCTA showed a significantly higher number of detectable nodes compared to DREAM OCTA (*p* = 0.0013; δ = − 0.80), while Triton OCTA exhibited a significantly lower number (*p* = 0.0001; δ = 0.93). No significant differences were detected between DREAM OCTA and Cirrus OCTA.

For fractal dimension (FD), DREAM OCTA measured a mean of 1.999 ± 0.003651, Spectralis OCTA 1.979 ± 0.00712, Triton OCTA 1.982 ± 0.00379, and Cirrus OCTA 1.98 ± 0.002626. DREAM OCTA exhibited a significantly larger FD compared to all three other devices (Spectralis: *p* < 0.0001; δ = 0.90; Triton: *p* < 0.0001; δ = 0.97; Cirrus: *p* < 0.0001; δ = 0.97).

The median vessel length (MVL) was also analysed, with DREAM OCTA measuring a median of 47 μm (IQR: 2 μm), Spectralis OCTA 41 μm (IQR: 2 μm), Triton OCTA 44 μm (IQR: 4 μm), and Cirrus OCTA 41 μm (IQR: 2 μm). DREAM OCTA presented a significantly larger MVL compared to Spectralis (*p* < 0.0001; δ = 1.00), Triton (*p* = 0.0307; δ = 0.70), and Cirrus (*p* < 0.0001; δ = 1.00).

**Fig. 4:**
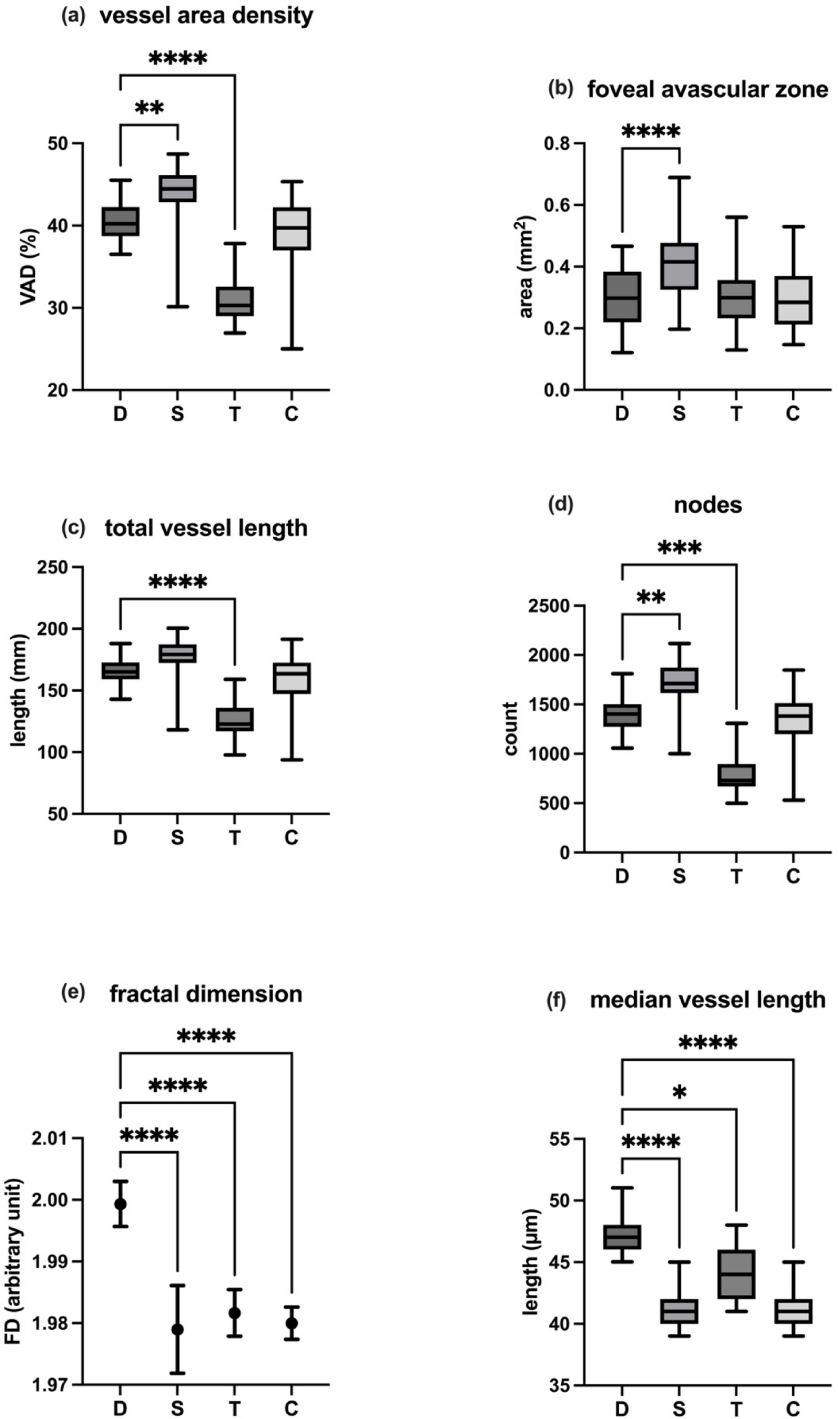
Results of the different image parameters for superficial capillary plexus derived from the single devices (D: DREAM; S: Spectralis; T: Triton; C: Cirrus). (**a**) vessel area density. (**b**) foveal avascular zone. (**c**) total vessel length. (**d**) number of nodes. (**e**) fractal dimension. (**f**) median vessel length. Significant *p*-values (< 0.05) of pairwise comparison with DREAM OCTA are marked by asterisks (with **** as *p* < 0.0001; *** as *p* < 0.001; ** as *p* < 0.01; * as *p* < 0.05).

All relevant data are presented in Table 2; Fig. 4, with errors reported as interquartile ranges when a Gaussian distribution was not present.

**Table 2:** Vascular parameters derived from the En face images of the different devices for superficial capillary plexus (SCP) and deep capillary plexus (DCP).

	DREAM (SCP)	Spectralis (SCP)	Triton (SCP)	Cirrus (SCP)	DREAM (DCP)	Spectralis (DCP)	Triton (DCP)	Cirrus (DCP)
VAD (%)	40.2	44.45	30.3	39.71	37.33	37.29	36.66	37.34
IQR_VAD_ (%)	3.50	3.30	3.59	5.22	1.64	2.85	1.86	5.26
FAZ (mm^2^)	0.298	0.416	0.299	0.2845	0.339	0.51	0.5935	0.9145
IQR_FAZ_(mm^2^)	0.164	0.1522	0.124	0.1575	0.2083	0.1867	0.257	0.3995
TVL (mm)	165.2	179.2	122.8	163.6	160.3	169.2	154.8	148.1
IQR_TVL_ (mm)	13.7	15	18.9	25.2	9.2	13.1	13.1	22.4
Nodes	1403	1711	729.5	1382	1413	1515	1309	1287
IQR_Nodes_	228	259	227	316	147	258	166	305
FD (mean)	1.999	1.979	1.982	1.98	1.98	1.981	1.98	1.98
SD_FD_	0.003651	0.00712	0.00379	0.002626	0.00391	0.006074	0.001826	0.000548
MVL (µm)	47	41	44	41				
IQR_MVL_ (µm)	2	2	4	2				
MVD (µm)					23	23	24	25
IQR_MVD_ (µm)					1	1.25	1	1
p (VAD)		**0.0019**	**< 0.0001**	> 0.9999		> 0.9999	**0.0416**	> 0.9999
p (FAZ)		**< 0.0001**	> 0.9999	> 0.9999		**0.0416**	**0.0004**	**< 0.0001**
p (TVL)		0.1287	**< 0.0001**	> 0.9999		0.1668	0.4312	**0.0307**
p (Nodes)		**0.0013**	**0.0001**	> 0.9999		> 0.9999	**0.0019**	**0.0162**
p (FD)		**< 0.0001**	**< 0.0001**	**< 0.0001**		> 0.9999	> 0.9999	> 0.9999
p (MVL)		**< 0.0001**	**0.0307**	**< 0.0001**				
p (MVD)						0.9691	**0.0069**	**0.0023**
δ (VAD)		− 0.87	1.00	0.00		0.13	0.53	0.07
δ (FAZ)		− 0.77	− 0.27	− 0.20		− 0.67	− 0.80	− 0.87
δ (TVL)		− 0.60	0.93	0.33		− 0.53	0.47	0.47
δ (Nodes)		− 0.80	0.93	− 0.07		− 0.33	0.80	0.47
δ (FD)		0.90	0.97	0.97		0.00	0.03	− 0.03
δ (MVL)		1.00	0.70	1.00				
δ (MVD)						0.33	− 0.60	− 0.63

Deviation given as interquartile range (IQR) or standard deviation (SD), respectively. *P*-values are given for pairwise comparison with the novel DREAM OCTA system. Effect size is given with Cliff’s delta (δ).

Significant *p*-values (*p* < 0.05) are reported in bold.

### Deep capillary plexus

The vessel area density (VAD) was measured as 37.33% (IQR: 1.64%) for DREAM OCTA, 37.29% (IQR: 2.85%) for Spectralis OCTA, 36.66% (IQR: 1.86%) for Triton OCTA, and 37.34% (IQR: 5.26%) for Cirrus OCTA. When comparing DREAM OCTA to the other devices, the VAD for Triton OCTA was significantly lower (*p* = 0.0416; δ = 0.53), while no significant differences were found between DREAM OCTA and the Spectralis or Cirrus devices.

Regarding the foveal avascular zone (FAZ), DREAM OCTA measured 0.339 mm^2^ (IQR: 0.2083 mm^2^), Spectralis OCTA 0.51 mm^2^ (IQR: 0.1867 mm^2^), Triton OCTA 0.5935 mm^2^ (IQR: 0.257 mm^2^), and Cirrus OCTA 0.9145 mm^2^ (IQR: 0.3995 mm^2^). FAZ was significantly smaller in DREAM OCTA en face images compared to all three other devices (Spectralis: *p* = 0.0416; δ = − 0.67; Triton: *p* = 0.0004; δ = − 0.80; Cirrus: *p* < 0.0001; δ = − 0.87).

For total vessel length (TVL), DREAM OCTA measured 160.3 mm (IQR: 9.2 mm), Spectralis OCTA 169.2 mm (IQR: 13.1 mm), Triton OCTA 154.8 mm (IQR: 13.1 mm), and Cirrus OCTA 148.1 mm (IQR: 22.4 mm). TVL was significantly lower in Cirrus OCTA compared to DREAM OCTA (*p* = 0.0307; δ = 0.47), while there were no significant differences between DREAM OCTA and the other two devices.

Regarding the number of nodes in the vascular architecture, DREAM OCTA detected 1413 nodes (IQR: 147), Spectralis OCTA 1515 nodes (IQR: 258), Triton OCTA 1309 nodes (IQR: 166), and Cirrus OCTA 1287 nodes (IQR: 305). Triton OCTA (*p* = 0.0019; δ = 0.80) and Cirrus OCTA (*p* = 0.0162; δ = 0.47) showed significantly fewer nodes compared to DREAM OCTA, while no significant difference was found between DREAM and Spectralis OCTA.

For the fractal dimension (FD), DREAM OCTA measured 1.98 ± 0.00391 (mean and standard deviation), Spectralis OCTA 1.981 ± 0.006074, Triton OCTA 1.98 ± 0.001826, and Cirrus OCTA 1.98 ± 0.000548. No statistically significant differences were observed between DREAM OCTA and each other device.

Finally, mean vessel diameter (MVD) was measured as 23 μm (IQR: 1 μm) for DREAM OCTA, 23 μm (IQR: 1.25 μm) for Spectralis OCTA, 24 μm (IQR: 1 μm) for Triton OCTA, and 25 μm (IQR: 1 μm) for Cirrus OCTA. MVD was significantly smaller in DREAM OCTA images compared to Triton OCTA (*p* = 0.0069; δ = − 0.60) and Cirrus OCTA (*p* = 0.0023; δ = − 0.63), with no significant difference observed between DREAM OCTA and Spectralis OCTA.

**Fig. 5:**
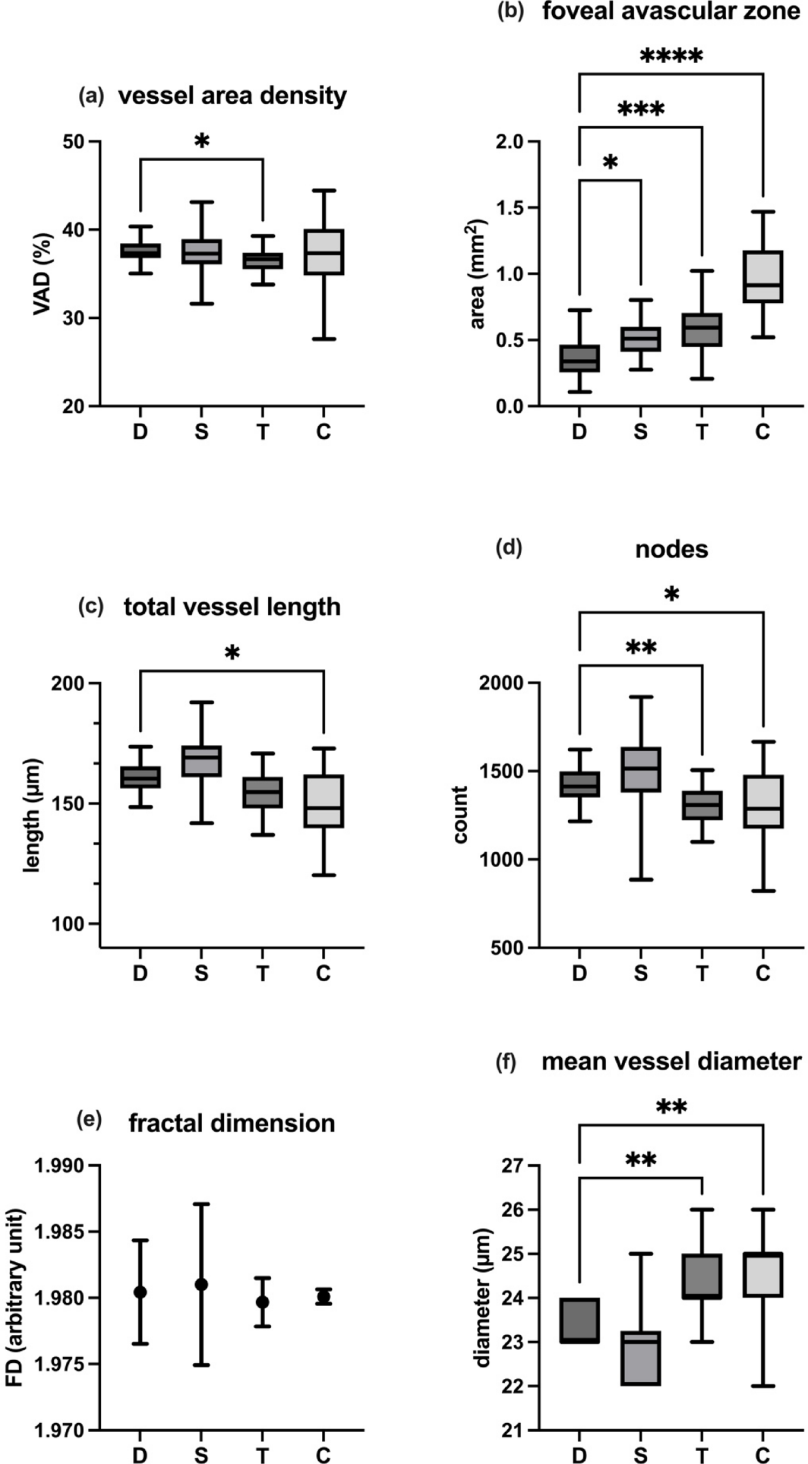
Results of the different image parameters for deep capillary plexus derived from the single devices (D: DREAM; S: Spectralis; T: Triton; C: Cirrus). (**a**) vessel area density. (**b**) foveal avascular zone. (**c**) total vessel length. (**d**) number of nodes. (**e**) fractal dimension. (**f**) mean vessel diameter. Significant p-values (< 0.05) of pairwise comparison with DREAM OCTA are marked by asterisks (with **** as *p* < 0.0001; *** as *p* < 0.001; ** as *p* < 0.01; * as *p* < 0.05).

All data are presented in Table 2; Fig. 5.

## Discussion

OCTA has become an essential, non-invasive imaging tool in clinical practice, providing high-resolution visualisation of retinal microvasculature^19^. With advancements in technology and a broad range of measurable parameters, OCTA holds great promise for accurately diagnosing and monitoring chorioretinal diseases.

In this study, we compared a novel SS-OCTA device (Intalight DREAM OCTA) with three well-established OCT systems (Heidelberg Spectralis, Topcon Triton, and Zeiss Cirrus 5000) in terms of acquisition time and multiple microvascular parameters in SCP and DCP. To ensure consistent metrics, we utilised the OCTAVA algorithm^13^ for automated and standardised cross-device en face image analysis. Particularly in the DCP, DREAM OCTA demonstrated superior vascular resolution. It outperformed both Triton and Cirrus OCTA, showing a higher number of nodes, a smaller mean vessel diameter, and a smaller foveal avascular zone (FAZ).

In the SCP, DREAM OCTA showed comparable results concerning the established OCTA systems, with more continuous vessel imaging (higher MVL) and greater complexity in the microvascular architecture (higher FD). For the DCP, DREAM OCTA provided high-quality images, particularly in the central area surrounding the FAZ, showing more precise vascular details and better small-vessel imaging compared to the other devices, matching Spectralis in quality and outperforming Triton and Cirrus.

A well-established parameter describing OCTA images is vessel area density (VAD). DREAM OCTA showed higher VAD values in SCP compared to Triton but lower than Spectralis, with no significant difference from Cirrus. In DCP, VAD for DREAM OCTA aligned with Spectralis and Cirrus but was higher than Triton. VAD was determined by measuring the area covered by vessel lumens after applying binary reconstruction to the images. While it provides a useful overview of the amount of retinal vascular structure detected, it offers limited information about vessel arrangement, continuity, and branching patterns. For that, it is helpful to consider further microvascular parameters for comparison.

The FAZ area is widely recognized as a clinical marker for monitoring retinal disease progression^20^. In our study, FAZ measurements in the SCP were consistent across DREAM, Triton, and Cirrus OCTA devices, though significantly larger in the Spectralis OCTA system. These findings suggest that the novel DREAM SS-OCTA shows strong alignment with established systems in terms of this well-established microvascular metric. However, the differences observed with the Spectralis system highlight the need to account for potential inter-device variations when comparing results from different studies. In the DCP, our analysis revealed significant differences in FAZ area across the OCT devices, with DREAM OCTA displaying the smallest FAZ area among the four systems. This suggests that DREAM OCTA provides a more precise measurement of the microvascular architecture, capturing the small vessels surrounding the FAZ more continuously and in greater detail, resulting in a reduced area without visible vessels. Figure 1 illustrates examples of FAZ imaging in DCP from each device, highlighting the variations in microvascular architecture captured around the FAZ.

To better characterize both the amount and the structural organization of retinal vessels, it is essential to examine additional microvascular parameters. When comparing TVL across devices, we found no statistically significant difference between DREAM OCTA and both Spectralis and Cirrus OCTA in the SCP, while TVL in Triton OCTA was significantly smaller. In the DCP, DREAM OCTA showed no statistically significant difference compared with Spectralis and Triton, but TVL in Cirrus OCTA was notably smaller. This parameter provides insight into each device’s ability to image and display both huge and small retinal vessels, with the OCTAVA algorithm applying a consistent threshold for vessel display quality and size^13^. A higher TVL indicates more precise imaging of the microvasculature, as more vessel length can be clearly distinguished from background noise.

The number of nodes identified in the skeletonized image serves as an indicator of how well different OCTA devices resolve the complex microvascular branching pattern. The DREAM OCTA demonstrated a significantly higher number of detected nodes in the SCP compared to the Triton, though fewer than the Spectralis device. No significant difference was observed between the DREAM and the Cirrus in SCP. In the DCP, the number of detectable nodes for the DREAM OCTA was comparable (no statistically significant difference was found) to the Spectralis OCTA and exceeded that of both the Triton and Cirrus OCTA devices.

The FD is a parameter quantifying the complexity of a vascular branching pattern^19^. In the SCP, DREAM OCTA showed a significantly higher FD compared to the other three devices, indicating its superior ability to capture and represent the complex intricate structure of retinal microvasculature. In contrast, no significant differences were observed in DCP. These findings suggest that DREAM OCTA is really good at precisely imaging the complex vessel networks in the SCP, outperforming the established devices in this aspect.

In the SCP, examining the MVL provides valuable insights into the continuity and consistency of the displayed vessels. DREAM OCTA demonstrated a significantly higher MVL compared to the other three devices, suggesting that it offers more continuous visualisation of retinal vessels with less background noise interfering with vessel branching. This highlights DREAM OCTA’s enhanced ability to capture and present retinal microvasculature more clearly than the established systems.

In DCP, it is particularly insightful to analyse MVD, as this parameter focuses on the diameter of the retinal vessels displayed. When compared to the Spectralis system, DREAM OCTA showed no significant difference in MVD. However, DREAM OCTA exhibited a significantly smaller MVD compared to Triton and Cirrus OCTA. This suggests that DREAM OCTA, like Spectralis, is capable of capturing and displaying small vessels in the DCP with high quality and detail, providing results ready for analysis, whereas Triton and Cirrus OCTA may be less precise in this regard.

One of the key advantages of the DREAM SS-OCT is its significantly shorter acquisition time for OCTA images, also due to its high scanning rate of 200 kHz^5^. Compared to the Spectralis OCT module with a scanning rate of 125 kHz^6^, which delivers almost comparable image quality in both the SCP and DCP, DREAM OCTA requires much less time for image acquisition: 9.125 s (IQR: 0.805 s) for DREAM OCTA versus 23.25 s (IQR: 10.43 s) for the Spectralis device. While the acquisition time for Cirrus OCTA is similar to that of DREAM OCTA (no statistically significant difference was found), DREAM provides superior image quality, particularly in the DCP. DREAM OCTA outperforms Triton OCTA in terms of acquisition speed (as well as image quality). Faster acquisition times are especially advantageous for patient comfort and adherence, particularly in older patients and in children who may struggle to remain still for extended periods of time. This becomes even more relevant in the presence of retinal pathologies, when visual acuity is frequently impaired and fixation stability reduced. Additionally, shorter acquisition times reduce motion artefacts, further enhancing overall image quality.

In conclusion, our study found that the novel DREAM SS-OCT provides en face OCTA images (3 mm x 3 mm) with good consistency when compared to three established OCT systems: Spectralis, Triton, and Cirrus.

In the context of the SCP, our findings indicate that DREAM OCTA demonstrates more continuous and refined imaging of retinal vessels, as evidenced by a higher MVL relative to the other devices, as well as a greater overall complexity of the microvascular architecture, reflected by a higher FD compared to the established systems.

In the DCP, DREAM OCTA produces images of high quality across the analysed parameters. Notably, in the central area surrounding the FAZ, this novel device provides more accurate information about the continuous vascular structure, as indicated by a smaller FAZ compared to established systems. Additionally, in the DCP, DREAM OCTA effectively captures small vessels and their branching patterns (higher number of nodes and lower MVD compared to Triton and Cirrus), achieving image quality that is at least comparable to Heidelberg Spectralis OCTA and surpassing the precision of Topcon Triton and Zeiss Cirrus OCTA.

DREAM OCTA enables high-resolution imaging of the microvascular architecture in the DCP, which holds significant promise for both clinical applications and future research, particularly for imaging deeper retinal layers. While the focus begins with DCP, it can potentially extend to areas such as choroidal neovascularization, choriocapillaris, and broader choroidal imaging. Additionally, the ability to visualise the vasculature of deeper retinal layers may facilitate the examination of patients with macular diseases characterised by thickening of the retinal layers above the vascular structures, for example, in cases of macular haemorrhage. This novel instrument may offer a more efficient and precise means of imaging associated choroidal neovascularization.

Improved visualization of the DCP may have significant clinical implications. A more precisely defined FAZ, along with enhanced vessel continuity, could facilitate the earlier detection of microvascular alterations characteristic of retinal diseases such as diabetic retinopathy^1^. Furthermore, conditions like Macular Telangiectasia Type II are associated with an enlargement of intervascular spaces and a reduction in capillary density in both the superficial and deep capillary plexuses^1^. Superior imaging of these subtle vascular changes may thus enable more accurate and timely diagnosis, allowing for earlier therapeutic intervention and potentially improving visual outcomes.

The overall high image quality, particularly in deeper retinal layers, combined with a short acquisition time, offers a valuable tool for gaining deeper insights into both retinal and systemic vascular pathologies.

An important limitation of this study is the exclusive inclusion of healthy, young (mean age of 26.41 ± 2.94 years) participants. While this design ensured optimal imaging conditions and minimized confounding factors, it inherently limits the generalizability of the findings to broader clinical populations. In patients with retinal disease, structural alterations and reduced fixation stability may affect both image acquisition and microvascular quantification. Future studies evaluating the DREAM OCTA system under real-world clinical conditions are therefore warranted. Another limitation of quantitative analysis beyond the built-in software lies in the varying pixel density of images acquired and exported by different instruments. To deal with this limitation and ensure consistent analysis as good as possible, we resized all images to the same dimensions, allowing for uniform application of image processing steps and filters across the dataset. Furthermore, although the use of OCTAVA enhances standardization of image analysis, segmentation of the FAZ, particularly in the DCP, remains vulnerable to artifacts. Variations in image quality, projection artifacts, and challenges in accurately defining vascular borders may influence FAZ measurements despite automated processing. This potential source of error should be considered when interpreting quantitative comparisons between devices.

## Conclusion

The findings of this study demonstrate that the DREAM OCTA device offers a promising advancement in retinal imaging, particularly for visualizing deep capillary plexus microvasculature. It provided superior vascular resolution, shown by higher median vessel length and fractal dimension in the superficial capillary plexus, as well as a higher number of nodes and a lower mean vessel diameter in the deep capillary plexus compared to established systems. Additionally, DREAM OCTA shows shorter acquisition time compared to Spectralis, without compromising image quality. Faster acquisition times improve patient comfort and adherence, especially for older patients and children who struggle to stay still. This can be crucial for retinal pathologies, where impaired visual acuity and fixation stability are common. Shorter times also minimize motion artifacts, improving image quality.

The most comparable measurements in vessel area density (VAD) and foveal avascular zone (FAZ) in the superficial capillary plexus further support its reliability (for VAD, Spectralis showed significantly higher and Triton significantly lower values; for FAZ, Spectralis showed significantly higher values). Overall, the DREAM OCTA shows strong potential for clinical and research applications in retinal and systemic vascular disease diagnostics. Future studies should focus on evaluating its performance across diverse patient populations and pathologies to validate these promising results.

## Data Availability

The datasets used and/or analysed during the current study are available upon reasonable request.
